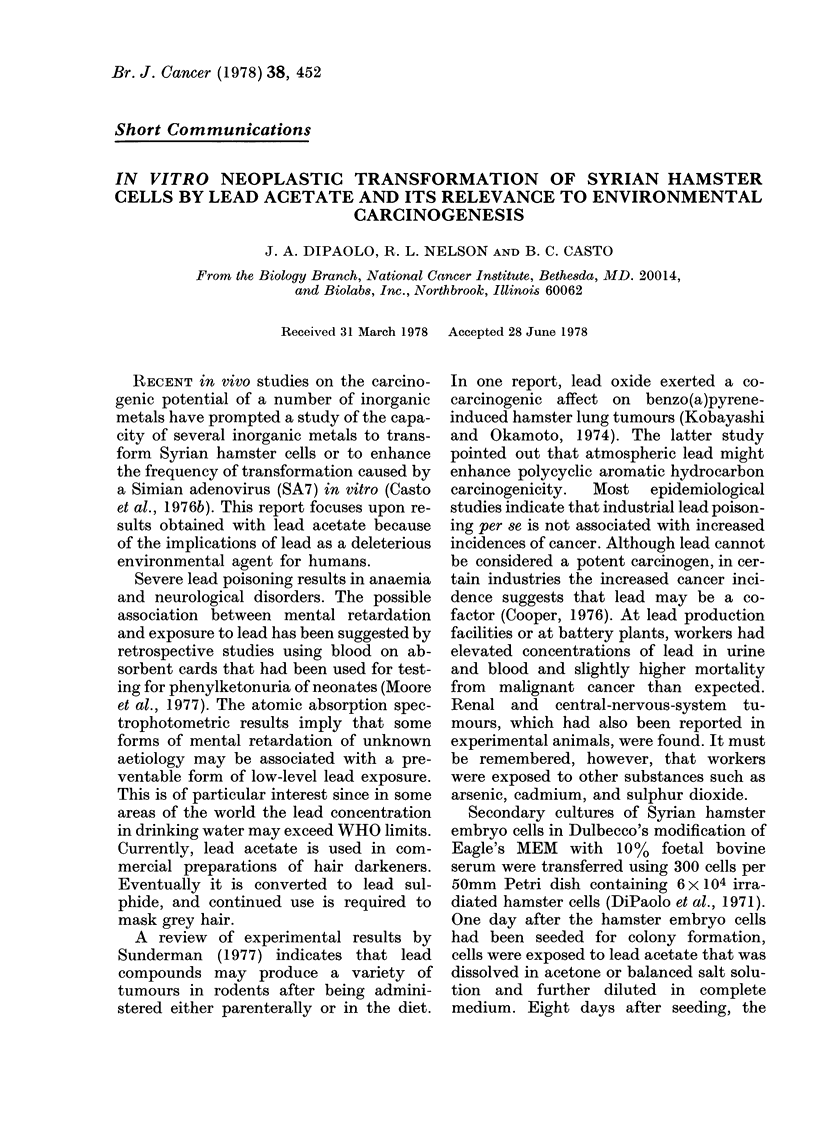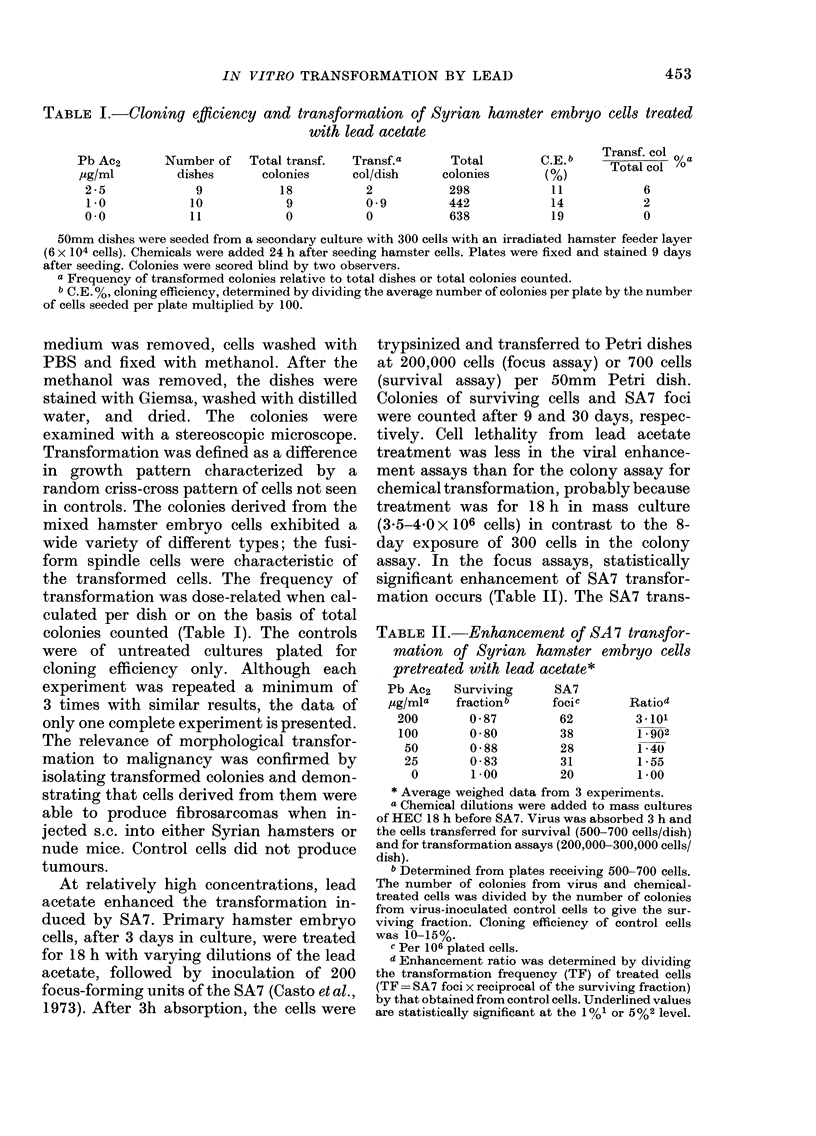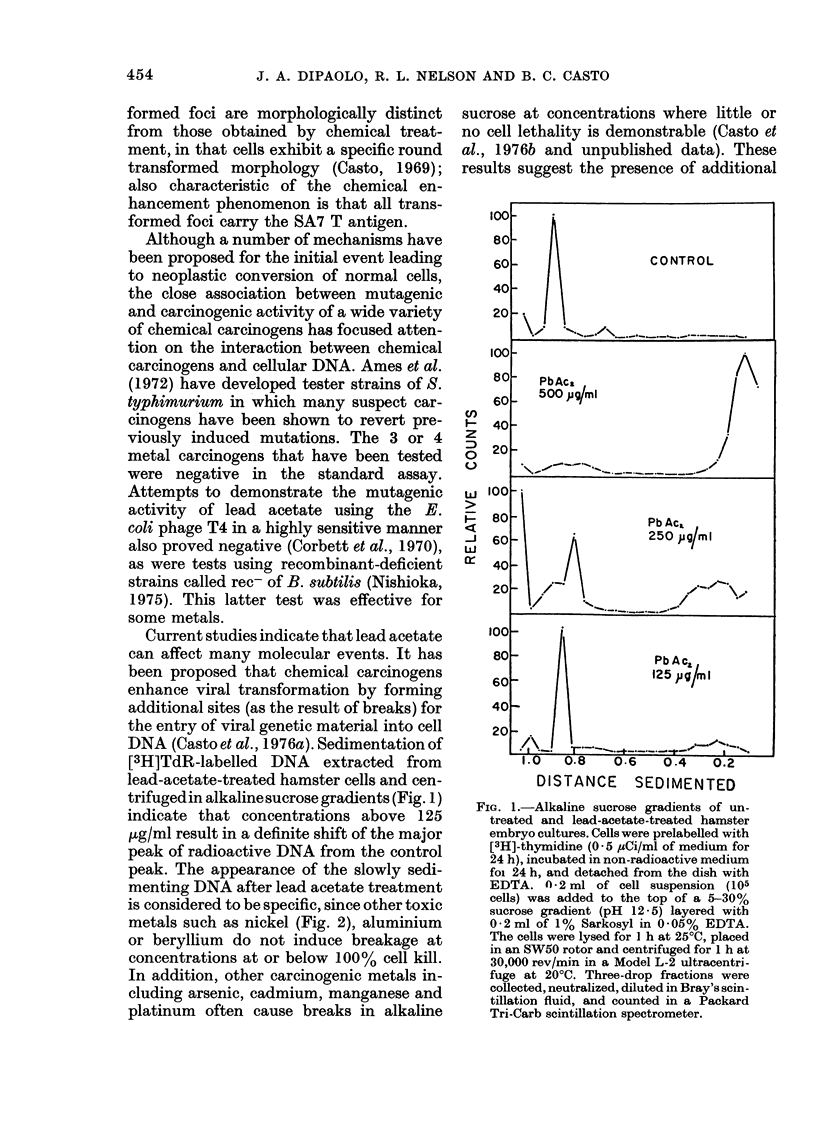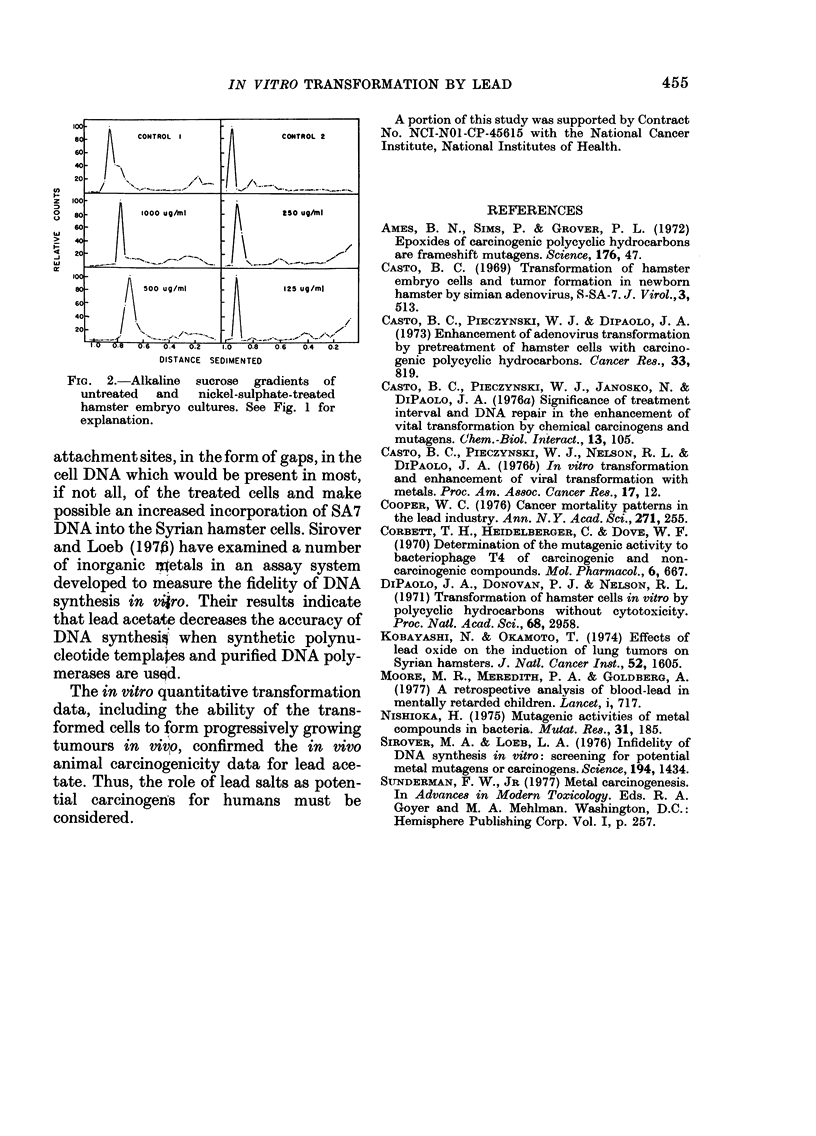# In vitro neoplastic transformation of Syrian hamster cells by lead acetate and its relevance to environmental carcinogenesis.

**DOI:** 10.1038/bjc.1978.228

**Published:** 1978-09

**Authors:** J. A. Dipaolo, R. L. Nelson, B. C. Casto


*Br. J. Cancer* (1978) **38**, 452

### *Short Communications*

### *IN VITRO* NEOPLASTIC TRANSFORMATION OF SYRIAN HAMSTER CELLS BY LEAD ACETATE AND ITS RELEVANCE TO ENVIRONMENTAL CARCINOGENESIS

J. A. DIPAOLO, R. L. NELSON AND B. C. CASTO

*From the Biology Branch, National Cancer Institute, Bethesda, MD.* 20014, *and Biolabs, Inc., Northbrook, Illinois* 60062

Received 31 March 1978 Accepted 28 June 1978

RECENT *in vivo* studies on the carcinogenic potential of a number of inorganic metals have prompted a study of the capacity of several inorganic metals to transform Syrian hamster cells or to enhance the frequency of transformation caused by a Simian adenovirus (SA7) *in vitro* (Casto *et al.*, 1976*b*). This report focuses upon results obtained with lead acetate because of the implications of lead as a deleterious environmental agent for humans.

Severe lead poisoning results in anaemia and neurological disorders. The possible association between mental retardation and exposure to lead has been suggested by retrospective studies using blood on absorbent cards that had been used for testing for phenylketonuria of neonates (Moore *et al.*, 1977). The atomic absorption spectrophotometric results imply that some forms of mental retardation of unknown aetiology may be associated with a preventable form of low-level lead exposure. This is of particular interest since in some areas of the world the lead concentration in drinking water may exceed WHO limits. Currently, lead acetate is used in commercial preparations of hair darkeners. Eventually it is converted to lead sulphide, and continued use is required to mask grey hair.

A review of experimental results by Sunderman (1977) indicates that lead compounds may produce a variety of tumours in rodents after being administered either parenterally or in the diet. In one report, lead oxide exerted a co-carcinogenic affect on benzo(a)pyrene-induced hamster lung tumours (Kobayashi and Okamoto, 1974). The latter study pointed out that atmospheric lead might enhance polycyclic aromatic hydrocarbon carcinogenicity. Most epidemiological studies indicate that industrial lead poisoning *per se* is not associated with increased incidences of cancer. Although lead cannot be considered a potent carcinogen, in certain industries the increased cancer incidence suggests that lead may be a co-factor (Cooper, 1976). At lead production facilities or at battery plants, workers had elevated concentrations of lead in urine and blood and slightly higher mortality from malignant cancer than expected. Renal and central-nervous-system tumours, which had also been reported in experimental animals, were found. It must be remembered, however, that workers were exposed to other substances such as arsenic, cadmium, and sulphur dioxide.

Secondary cultures of Syrian hamster embryo cells in Dulbecco's modification of Eagle's MEM with 10% foetal bovine serum were transferred using 300 cells per 50mm Petri dish containing $6 \times 10^4$ irradiated hamster cells (DiPaolo *et al.*, 1971). One day after the hamster embryo cells had been seeded for colony formation, cells were exposed to lead acetate that was dissolved in acetone or balanced salt solution and further diluted in complete medium. Eight days after seeding, the

TABLE I.—*Cloning efficiency and transformation of Syrian hamster embryo cells treated with lead acetate*

| Pb $Ac_2$ μg/ml | Number of dishes | Total transf. colonies | Transf.[a] col/dish | Total colonies | C.E.[b] (%) | $\frac{\text{Transf. col}}{\text{Total col}}$ %[a] |
|---|---|---|---|---|---|---|
| 2·5 | 9 | 18 | 2 | 298 | 11 | 6 |
| 1·0 | 10 | 9 | 0·9 | 442 | 14 | 2 |
| 0·0 | 11 | 0 | 0 | 638 | 19 | 0 |

50mm dishes were seeded from a secondary culture with 300 cells with an irradiated hamster feeder layer ($6 \times 10^4$ cells). Chemicals were added 24 h after seeding hamster cells. Plates were fixed and stained 9 days after seeding. Colonies were scored blind by two observers.

[a] Frequency of transformed colonies relative to total dishes or total colonies counted.

[b] C.E.%, cloning efficiency, determined by dividing the average number of colonies per plate by the number of cells seeded per plate multiplied by 100.

medium was removed, cells washed with PBS and fixed with methanol. After the methanol was removed, the dishes were stained with Giemsa, washed with distilled water, and dried. The colonies were examined with a stereoscopic microscope. Transformation was defined as a difference in growth pattern characterized by a random criss-cross pattern of cells not seen in controls. The colonies derived from the mixed hamster embryo cells exhibited a wide variety of different types; the fusiform spindle cells were characteristic of the transformed cells. The frequency of transformation was dose-related when calculated per dish or on the basis of total colonies counted (Table I). The controls were of untreated cultures plated for cloning efficiency only. Although each experiment was repeated a minimum of 3 times with similar results, the data of only one complete experiment is presented. The relevance of morphological transformation to malignancy was confirmed by isolating transformed colonies and demonstrating that cells derived from them were able to produce fibrosarcomas when injected s.c. into either Syrian hamsters or nude mice. Control cells did not produce tumours.

At relatively high concentrations, lead acetate enhanced the transformation induced by SA7. Primary hamster embryo cells, after 3 days in culture, were treated for 18 h with varying dilutions of the lead acetate, followed by inoculation of 200 focus-forming units of the SA7 (Casto *et al.*, 1973). After 3h absorption, the cells were trypsinized and transferred to Petri dishes at 200,000 cells (focus assay) or 700 cells (survival assay) per 50mm Petri dish. Colonies of surviving cells and SA7 foci were counted after 9 and 30 days, respectively. Cell lethality from lead acetate treatment was less in the viral enhancement assays than for the colony assay for chemical transformation, probably because treatment was for 18 h in mass culture ($3{\cdot}5$–$4{\cdot}0 \times 10^6$ cells) in contrast to the 8-day exposure of 300 cells in the colony assay. In the focus assays, statistically significant enhancement of SA7 transformation occurs (Table II). The SA7 trans-

TABLE II.—*Enhancement of SA7 transformation of Syrian hamster embryo cells pretreated with lead acetate**

| Pb $Ac_2$ μg/ml[a] | Surviving fraction[b] | SA7 foci[c] | Ratio[d] |
|---|---|---|---|
| 200 | 0·87 | 62 | 3·10[1] |
| 100 | 0·80 | 38 | 1·90[2] |
| 50 | 0·88 | 28 | 1·40 |
| 25 | 0·83 | 31 | 1·55 |
| 0 | 1·00 | 20 | 1·00 |

* Average weighed data from 3 experiments.

[a] Chemical dilutions were added to mass cultures of HEC 18 h before SA7. Virus was absorbed 3 h and the cells transferred for survival (500–700 cells/dish) and for transformation assays (200,000–300,000 cells/dish).

[b] Determined from plates receiving 500–700 cells. The number of colonies from virus and chemical-treated cells was divided by the number of colonies from virus-inoculated control cells to give the surviving fraction. Cloning efficiency of control cells was 10–15%.

[c] Per $10^6$ plated cells.

[d] Enhancement ratio was determined by dividing the transformation frequency (TF) of treated cells (TF = SA7 foci × reciprocal of the surviving fraction) by that obtained from control cells. Underlined values are statistically significant at the 1%[1] or 5%[2] level.

formed foci are morphologically distinct from those obtained by chemical treatment, in that cells exhibit a specific round transformed morphology (Casto, 1969); also characteristic of the chemical enhancement phenomenon is that all transformed foci carry the SA7 T antigen.

Although a number of mechanisms have been proposed for the initial event leading to neoplastic conversion of normal cells, the close association between mutagenic and carcinogenic activity of a wide variety of chemical carcinogens has focused attention on the interaction between chemical carcinogens and cellular DNA. Ames *et al.* (1972) have developed tester strains of *S. typhimurium* in which many suspect carcinogens have been shown to revert previously induced mutations. The 3 or 4 metal carcinogens that have been tested were negative in the standard assay. Attempts to demonstrate the mutagenic activity of lead acetate using the *E. coli* phage T4 in a highly sensitive manner also proved negative (Corbett *et al.*, 1970), as were tests using recombinant-deficient strains called rec$^-$ of *B. subtilis* (Nishioka, 1975). This latter test was effective for some metals.

Current studies indicate that lead acetate can affect many molecular events. It has been proposed that chemical carcinogens enhance viral transformation by forming additional sites (as the result of breaks) for the entry of viral genetic material into cell DNA (Casto *et al.*, 1976*a*). Sedimentation of [$^3$H]TdR-labelled DNA extracted from lead-acetate-treated hamster cells and centrifuged in alkaline sucrose gradients (Fig. 1) indicate that concentrations above 125 $\mu$g/ml result in a definite shift of the major peak of radioactive DNA from the control peak. The appearance of the slowly sedimenting DNA after lead acetate treatment is considered to be specific, since other toxic metals such as nickel (Fig. 2), aluminium or beryllium do not induce breakage at concentrations at or below 100% cell kill. In addition, other carcinogenic metals including arsenic, cadmium, manganese and platinum often cause breaks in alkaline sucrose at concentrations where little or no cell lethality is demonstrable (Casto *et al.*, 1976*b* and unpublished data). These results suggest the presence of additional

FIG. 1.—Alkaline sucrose gradients of untreated and lead-acetate-treated hamster embryo cultures. Cells were prelabelled with [$^3$H]-thymidine (0·5 $\mu$Ci/ml of medium for 24 h), incubated in non-radioactive medium foı 24 h, and detached from the dish with EDTA. 0·2 ml of cell suspension ($10^5$ cells) was added to the top of a 5–30% sucrose gradient (pH 12·5) layered with 0·2 ml of 1% Sarkosyl in 0·05% EDTA. The cells were lysed for 1 h at 25°C, placed in an SW50 rotor and centrifuged for 1 h at 30,000 rev/min in a Model L-2 ultracentrifuge at 20°C. Three-drop fractions were collected, neutralized, diluted in Bray's scintillation fluid, and counted in a Packard Tri-Carb scintillation spectrometer.

FIG. 2.—Alkaline sucrose gradients of untreated and nickel-sulphate-treated hamster embryo cultures. See Fig. 1 for explanation.

attachment sites, in the form of gaps, in the cell DNA which would be present in most, if not all, of the treated cells and make possible an increased incorporation of SA7 DNA into the Syrian hamster cells. Sirover and Loeb (1976) have examined a number of inorganic metals in an assay system developed to measure the fidelity of DNA synthesis *in vitro*. Their results indicate that lead acetate decreases the accuracy of DNA synthesis when synthetic polynucleotide templates and purified DNA polymerases are used.

The *in vitro* quantitative transformation data, including the ability of the transformed cells to form progressively growing tumours *in vivo*, confirmed the *in vivo* animal carcinogenicity data for lead acetate. Thus, the role of lead salts as potential carcinogens for humans must be considered.

A portion of this study was supported by Contract No. NCI-N01-CP-45615 with the National Cancer Institute, National Institutes of Health.

### REFERENCES

AMES, B. N., SIMS, P. & GROVER, P. L. (1972) Epoxides of carcinogenic polycyclic hydrocarbons are frameshift mutagens. *Science*, **176**, 47.

CASTO, B. C. (1969) Transformation of hamster embryo cells and tumor formation in newborn hamster by simian adenovirus, S-SA-7. *J. Virol.*, **3**, 513.

CASTO, B. C., PIECZYNSKI, W. J. & DIPAOLO, J. A. (1973) Enhancement of adenovirus transformation by pretreatment of hamster cells with carcinogenic polycyclic hydrocarbons. *Cancer Res.*, **33**, 819.

CASTO, B. C., PIECZYNSKI, W. J., JANOSKO, N. & DIPAOLO, J. A. (1976*a*) Significance of treatment interval and DNA repair in the enhancement of vital transformation by chemical carcinogens and mutagens. *Chem.-Biol. Interact.*, **13**, 105.

CASTO, B. C., PIECZYNSKI, W. J., NELSON, R. L. & DIPAOLO, J. A. (1976*b*) *In vitro* transformation and enhancement of viral transformation with metals. *Proc. Am. Assoc. Cancer Res.*, **17**, 12.

COOPER, W. C. (1976) Cancer mortality patterns in the lead industry. *Ann. N.Y. Acad. Sci.*, **271**, 255.

CORBETT, T. H., HEIDELBERGER, C. & DOVE, W. F. (1970) Determination of the mutagenic activity to bacteriophage T4 of carcinogenic and non-carcinogenic compounds. *Mol. Pharmacol.*, **6**, 667.

DIPAOLO, J. A., DONOVAN, P. J. & NELSON, R. L. (1971) Transformation of hamster cells *in vitro* by polycyclic hydrocarbons without cytotoxicity. *Proc. Natl. Acad. Sci.*, **68**, 2958.

KOBAYASHI, N. & OKAMOTO, T. (1974) Effects of lead oxide on the induction of lung tumors on Syrian hamsters. *J. Natl. Cancer Inst.*, **52**, 1605.

MOORE, M. R., MEREDITH, P. A. & GOLDBERG, A. (1977) A retrospective analysis of blood-lead in mentally retarded children. *Lancet*, i, 717.

NISHIOKA, H. (1975) Mutagenic activities of metal compounds in bacteria. *Mutat. Res.*, **31**, 185.

SIROVER, M. A. & LOEB, L. A. (1976) Infidelity of DNA synthesis *in vitro*: screening for potential metal mutagens or carcinogens. *Science*, **194**, 1434.

SUNDERMAN, F. W., JR (1977) Metal carcinogenesis. In *Advances in Modern Toxicology*. Eds. R. A. Goyer and M. A. Mehlman. Washington, D.C.: Hemisphere Publishing Corp. Vol. I, p. 257.